# Personal

**Published:** 1915-11

**Authors:** 


### PERSONAL.

**Announcement of removal, travel, and other matters of interest are requested. Please report errors in the listing of any physician in the State and other directories, that we may co-operate with the State Society in securing a correct list.**

---

Dr. M. J. Wilson of Warsaw visited in Lowell, Mass., in October.

---

Dr. C. A. Brownell of Buffalo is about to move to Jewettville.

---

Dr. David Wheeler of Buffalo, who joined the American Ambulance Service early in the European War, subsequently entered the Foreign Legion of France. On Sept. 28, he was wounded in an engagement and, in spite of a shattered leg, crawled 7 miles to a place of safety, attending to the needs of other wounded soldiers, as he went. It was at first thought that the leg would require amputation but later reports indicate that this will not be necessary. It is no breach of neutrality to respect the sentiment of love for an ancestral country and the heroism displayed in voluntary service with its allies.

---

Dr. Horace H. Le Seur of Niagara Falls, son of Dr. J. W. Le Seur of Batavia, was married to Miss Minerva Crossett, at Batavia, Sept. 29. Dr. and Mrs. Le Seur will reside at 711 Townsend Place, Niagara Falls.

---

Dr. F. W. Welch of Buffalo, has been appointed Sanitary Inspector at a salary of $1000.

---

Dr. James C. Haley of Buffalo has been promoted from Assistant Medical School Examiner, to Sanitary Inspector at $1000.

